# Hemodynamic and clinical onset in patients with hereditary pulmonary arterial hypertension and *BMPR2 *mutations

**DOI:** 10.1186/1465-9921-12-99

**Published:** 2011-07-29

**Authors:** Nicole Pfarr, Justyna Szamalek-Hoegel, Christine Fischer, Katrin Hinderhofer, Christian Nagel, Nicola Ehlken, Henning Tiede, Horst Olschewski, Frank Reichenberger, Ardeschir HA Ghofrani, Werner Seeger, Ekkehard Grünig

**Affiliations:** 1 Centre for Pulmonary Hypertension Thoraxclinic, University of Heidelberg, Heidelberg, Germany; 2 Institute of Human Genetics, University of Heidelberg, Germany; 3 University of Giessen Lung Centre, Giessen, Germany; 4 Department of Pneumology, University of Graz, Graz, Austria

## Abstract

**Background:**

Mutations in the *bone morphogenetic protein receptor 2 *(*BMPR2*) gene can lead to idiopathic pulmonary arterial hypertension (IPAH). This study prospectively screened for *BMPR2 *mutations in a large cohort of PAH-patients and compared clinical features between *BMPR2 *mutation carriers and non-carriers.

**Methods:**

Patients have been assessed by right heart catheterization and genetic testing. In all patients a detailed family history and pedigree analysis have been obtained. We compared age at diagnosis and hemodynamic parameters between carriers and non-carriers of *BMPR2 *mutations. In non-carriers with familial aggregation of PAH further genes/gene regions as the *BMPR2 *promoter region, the *ACVRL1*, *Endoglin*, and *SMAD8 *genes have been analysed.

**Results:**

Of the 231 index patients 22 revealed a confirmed familial aggregation of the disease (HPAH), 209 patients had sporadic IPAH. In 49 patients (86.3% of patients with familial aggregation and 14.3% of sporadic IPAH) mutations of the *BMPR2 *gene have been identified. Twelve *BMPR2 *mutations and 3 unclassified sequence variants have not yet been described before. Mutation carriers were significantly younger at diagnosis than non-carriers (38.53 ± 12.38 vs. 45.78 ± 11.32 years, p < 0.001) and had a more severe hemodynamic compromise. No gene defects have been detected in 3 patients with HPAH.

**Conclusion:**

This study identified in a large prospectively assessed cohort of PAH- patients new *BMPR2 *mutations, which have not been described before and confirmed previous findings that mutation carriers are younger at diagnosis with a more severe hemodynamic compromise. Thus, screening for *BMPR2 *mutations may be clinically useful.

## Introduction

Pulmonary arterial hypertension (PAH) is a rare vascular disorder characterised by increased pulmonary vascular resistance and right heart failure. PAH can be idiopathic (IPAH), heritable (HPAH) or associated with other conditions (APAH) as connective tissue diseases, congenital heart diseases, portal hypertension, drug or toxin exposure [1,2]. Heterozygous germline mutations in the *bone morphogenetic protein type 2 receptor *(*BMPR2*) have been identified as a gene underlying HPAH in approximately 10 to 40% of patients with apparently sporadic disease [1,3-6] and in 58% to 74% of patients with familial PAH [1,4,6,7]. In total 298 different mutations in *BMPR2 *have been identified so far in independent patients including those with a known PAH family history, sporadic disease and PAH associated with other diseases [1]. In a few PAH patients mutations in other genes participating in the BMPR2 signalling pathway have been identified, as *Activin A receptor type II-like 1 *(*ACVRL1*, also called *ALK1*) [8], *Endoglin *[9], and *SMAD8 *[10]. Nevertheless, there is still a small proportion of patients with familial aggregation of PAH in which no gene defects can be detected so far [7,11].

HPAH patients carrying a *BMPR2 *mutation develop the disease approximately 10 years earlier than non-carriers, with more severe hemodynamic changes [6,12-15] and a reduced response to acute vasodilator testing [6,12,14-16]. Patients carrying *ACVRL1 or Endoglin *mutations have been characterised to be of younger age at diagnosis and death as patients without mutations [14]. A recent study of Austin et al [17] showed that HPAH female patients with missense mutations in the *BMPR2 *gene had a more severe disease than patients with truncating mutations. These publications indicate that the clinical phenotype of PAH can be affected by the type of mutation. However, most data comparing clinical features between *BMPR2 *mutation carriers and non-carriers have been obtained from registries as from the French Network of Pulmonary Hypertension [6,13-15], and from centres in the United States as the New York Presbyterian Pulmonary Hypertension Center [12], the Utah Pulmonary Hypertension Genetics Project [16] or the Vanderbilt University School of Medicine, Nashville, Tennessee [7,17,18] and are retrospective in design. The genetic mechanism of PAH remains unclear in those families in which no *BMPR2 *mutation can be detected.

Therefore, the aim of this study was to evaluate hemodynamic parameters and genetic status in a large German cohort of patients using a prospective design. The frequency of known *BMPR2 *mutations has been analysed and a detailed search for new *BMPR2 *mutations has been performed. In this study, we present 12 new *BMPR2 *mutations and 3 unclassified variants which have not been described before. Furthermore, we describe the clinical features of families with confirmed familial aggregation of PAH but no detectable mutations of the *BMPR2 *gene and tested these families for mutations of the genes *ACVRL1*, *Endoglin*, and *SMAD8*.

## Materials and methods

### Study Population

This prospective study investigated adult patients (≥ 18 years) with confirmed sporadic IPAH or familial HPAH between January 2006 and December 2009, who agreed to a genetic testing and from whom EDTA-blood was obtained. Patients have been seen in the centres of pulmonary hypertension (PH) of Heidelberg and Giessen and underwent complete clinical and genetic work-up. In all patients a right heart catheterization and a detailed family history was obtained and a three to four generation pedigree was constructed. For deceased relatives, medical records were reviewed when available and the diagnosis of PAH was based on the criteria used for index patients as well as on the results of the post mortem examination. Familial disease has been postulated when PAH was diagnosed in at least two family members. Sporadic IPAH was stated when family history and medical records of family members were negative.

The Ethics Committees of the Medical Faculties of the Universities of Heidelberg and Giessen approved the protocol of this study, and the family members gave their written informed consent. All participating patients and family members underwent genetic counselling. The study was part of the European Projects "Pulmotension" which belongs to the 6th European Framework.

### Mutation analysis of the *BMPR2 *gene

EDTA-blood samples were collected for genetic analysis in all patients and from all family members, if available. Human genomic DNA was prepared from peripheral blood lymphocytes. The complete coding sequence and exon/intron boundaries of the *BMPR2 *gene from each individual were amplified and analysed by DHPLC and/or direct sequencing as previously described [4]. HPAH patients without an obvious *BMPR2 *mutation were also analysed for mutations in the *BMPR2 *promoter, the *ACVRL1 *gene, *Endoglin *gene, and *SMAD8 *gene. In HPAH cases all first degree relatives were investigated for the mutation identified in the index patient. Primer sequences and PCR conditions are available upon request. Standard DNA sequencing reactions were performed using version 1.1 of Big Dye terminator cycle sequencing kit (Applied Biosystems Inc., Darmstadt) and were analysed on a Genetic Analyzer 3100 (Applied Biosystems Inc., Darmstadt). Pathogenicity of identified sequence alterations were assessed by use of the program MutationTaster http://www.mutationtaster.org/ and by ESEfinder 3.0 software http://rulai.cshl.edu/cgi-bin/tools/ESE3/esefinder.cgi.

Screening for larger rearrangements was performed with the SALSA Multiplex Ligation-dependent Probe Amplification (MLPA) P093-B1 HHT/PPH1 probe mix kit (MRC-Holland BV, Amsterdam, The Netherlands).

The mutation nomenclature refers to the NCBI human *BMPR2 *nucleotide sequence (NCBI: NM_001204) and is expressed following the standard recommendations of the Association for Molecular Pathology Training and Education Committee [19] with the A of the ATG start codon denoted as +1 and the initiator methionine as codon 1.

## Results

### Study Population

Between January 2006 and December 2009 in total 262 patients agreed to participate in the study and EDTA-blood has been stored for genetic analysis. Thirty-one patients had to be excluded due to several reasons. In 23 patients the further diagnostic work-up revealed a non-idiopathic form of pulmonary hypertension. In 3 patients the clinical data have been incomplete and in another 5 patients not enough blood for genetic analysis has been obtained. Thus, the study group for a complete genetic work-up consisted of 231 patients. All investigated patients were of Caucasian origin. About 91% of the analysed patients included in this study were of German ancestry, 4.8% were sent from different European countries (as Spain, Belgium, Netherlands, Sweden, Italy, and Eastern Europe), 1.3% were of Arabian ancestry and 2.6% of Turkish ancestry.

### Genetic disposition to PAH in the study population

**Figure 1:**
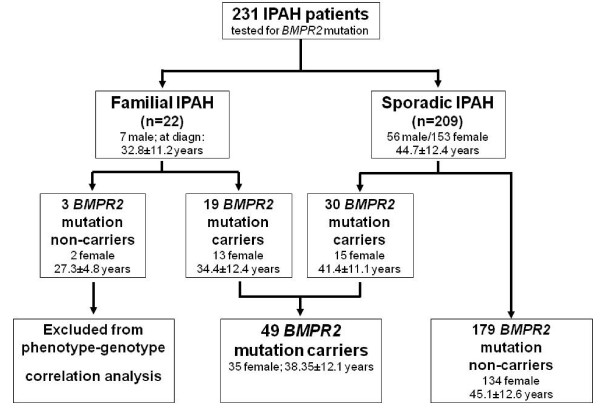
**Genetic disposition of the study population**. PAH = pulmonary arterial hypertension, IPAH = idiopathic PAH. The figure shows the proportion of *BMPR2 *mutation carriers in the study population, female to male proportion and the mean age at diagnosis.

Of the 231 PAH index patients 22 (9.5%) revealed a confirmed familial aggregation of the disease with at least one further affected family member. The remaining 209 patients (90.5%) with negative family history have been classified as sporadic IPAH cases (Figure 1). In 49 patients of the 231 PAH index patients (21.2%) including 19 of the 22 familial (86.4%) and in 30 of the 209 (14.4%) apparently sporadic cases, mutations in the *BMPR2 *gene have been identified (Figure 1).

### Clinical and hemodynamic characteristics

The mean age at diagnosis of all 231 patients was 43.49 ± 12.75 years; 168 patients were females reflecting a female to male ratio of 2.7:1. *BMPR2 *mutation carriers were significantly younger at diagnosis than non-carriers (Table 1). In three families without any identified mutation in *BMPR2*, *ACVRL1*, *ENG*, or *SMAD8 *the mean age at diagnosis (27.3 y ± 4.78) was significantly lower than that of the mutation carriers and non-carriers (HPAH: 38.53 y ± 12.38 and IPAH: 45.78 y ± 11.32, p < 0.01), respectively. Since the mutation carrier status could not be clarified in these families they have been excluded for the genotype-phenotype comparison (Figure 1). Gender distribution was slightly but not significantly different in the mutation carriers (female/male ratio 1.9:1) and non-carriers (ratio 3:1, table 1).

**Table 1:** Clinical characteristics at diagnosis

	*t-test*	n = 228 43.49 y ± 12.75			
***Patients***		**Mutation carrier** **n = 49**		**Mutation non carrier** **n = 179**	

*Age at onset (years)*	**	38.53 y ± 12.38		45.78 y ± 11.32	
*female/male (ratio)*		32/17 (1.9:1)		134/45 (3:1)	
*NYHA at diagnosis*		III-IV		II-IV	

*Pulmonary hemodynamic parameters*					

*Heart rate per minute*	*	83.57 ± 11.79	n = 28	77.38 ± 9.07	n = 88

*SaO^2 ^(%)*		92.85 ± 3.06	n = 26	92.68 ± 3.29	n = 83

*PASP (mm Hg)*	*	98.5 ± 16.35	n = 26	87.73 ± 18.78	n = 83

*PADP (mm Hg)*		44.5 ± 7.5	n = 26	36.37 ± 9.22	n = 81

*mPAP (mm Hg)*	***	62.63 ± 9.92	n = 35	53.44 ± 12.18	n = 135

*PCWP*		7.08 ± 3.16	n = 26	7.75 ± 2.42	n = 83

*SASP (mm Hg)*	*	118.11 ± 14.34	n = 27	128.13 ± 18.59	n = 86

*SADP (mm Hg)*		76.5 ± 11.81	n = 27	76.67 ± 10.60	n = 86

*CI (Litres/min/m^2^)*	***	1.67 ± 0.25	n = 31	2.10 ± 0.53	n = 124

*PVRI*	***	2306.53 ± 770.33	n = 25	1503.25 ± 671.76	n = 116

*PVR*	***	1519.65 ± 374.65	n = 22	1000.36 ± 456.51	n = 71

* < 0.05

** < 0.01

*** < 0.005

*BMPR2 *mutation carriers had a significantly higher mean pulmonary artery pressure (mPAP) and pulmonary vascular resistance (PVR), and a significantly lower cardiac index (CI) than non-carriers (Table 1). Both groups did not significantly differ in WHO-functional class, oxygen saturation, heart rate, pulmonary capillary wedge pressures (PCWP), and systemic arterial systolic (SASP) and diastolic (SADP) blood pressures (Table 1).

No correlation was seen in our data between truncating or missense mutation and sex, age of onset, and hemodynamic measurements (data not shown).

### *BMPR2 *mutations (Table 2)

**Table 2:** Details of *BMPR2 *mutations

Patient	new	Mutation Location Exon	Nucleotide Change	Amino Acid Change	Mutation type	Age at diagnosis
K6628		1	c.?_-540_76_?del	Del aa1-25?	Deletion	50 y
K4808		1	c.?_-540_76_?del	Del aa1-25?	Deletion	23 y
K9063		1	c.48G > A	p.W16X	Nonsense	14 y
K4518	*	2	c.91G > T	p.E31X	Nonsense	45 y
K4452	*	2	c.244C > T	p.Q82X	Nonsense	39 y
K1893	*	2-3	Del c.77?-c.418?		Deletion	27 y
K7369		3	c.353C > T	p.C118Y	Missense	56 y
K15016		3	c.377A > G	p.N126S	Missense	28 y
K14629		3	c.377A > G	p.N126S	Missense	61 y
K7341		3	c.?_248-c.418_?del		Deletion	31 y
K2878	*	Intron 3	c.418+5G > A		Splice defect	25 y
K14983		4	c.439C > T	p.R147X	Nonsense	49 y
K6834	*	4	c.461C > G	p.A154G	Missense/unclassified variant	33 y
K7833		4	c.507 C > A	p.C169X	Nonsense	41 y
K2917	*	4-13	Del c.419? - c.3017?		Deletion	30 y
K6565		6	c.631G > A	p.R211X	Nonsense	51 y
K6686	*	6	c.660insG	p. G220fsX224	Frameshift	18 y
K14147		6	c.818T > G	p.M273R	Missense	59 y
K5429		7	c.961C > T	p.R321X	Nonsense	27 y
K5633		7	c.961C > T	p.R321X	Nonsense	50 y
K12665		7	c.961C > T	p.R321X	Nonsense	69 y
K3771		Intron 8	c.1128+1G > T	del aa323-425	Splice defect	40 y
K7892	*	9	c.1157A > G	p.E386G	Missense/unclassified variant	52 y
K8027		9	c.1259G > A	p.C420Y	Missense	56 y
K11314		9	c.1258T > C	p.C420R	Missense	28 y
K15582	*	10	c.1296C > G	p.Y432X	Nonsense	28 y
K15529		10	c.1297C > T	p.Q433X	Nonsense	32 y
K4690		10	c.1313-1316delCAGA	p.T438fsX472	Frameshift	43 y
MHH09		10	c.1348C > T	p.Q450X	Nonsense	44 y
MHH52		10	c.1388insA	p.P463fsX470	Frameshift	52 y
K5943		10	c.1397G > A	p.W466X	Nonsense	47 y
K14763	*	Intron 10	c.1413+1G > A		Splice defect	43 y
K7816		Intron 10	c.1413+3A > T	p.G426fsX453	Splice defect	45 y
K12666	*	11	c.1460A > T	p.D487V	Missense/unclassified variant	42 y
K6717		11	c.1471C > T	p.R491W	Missense	70 y
K6361		11	c.1471C > T	p.R491W	Missense	40 y
K6201		11	c.1471C > T	p.R491W	Missense	30 y
K11744		11	c.1472G > A	p.R491Q	Missense	26 y
K5590		11	c.1483C > T	p.Q495X	Nonsense	35 y
K7936	*	11	c.1523G > A	p.W508X	Nonsense	40 y
K13356		11-12	Del c.1414-? _2866+?		Deletion	17.25 y
K10005	*	12	c.1598A > G	p.H533R	Missense	26 y
MHH18		12	c.1750C > T	p.R584X	Nonsense	62 y
K14424	*	12	c.2308delC	p.R770fsX771	Frameshift	29 y
K12298		12	c.2617C > T	p.R873X	Nonsense	50 y
K12921		12	c.2617C > T	p.R873X	Nonsense	53 y
K13213	*	12	c.2626C > T	p.Q876X	Nonsense	26 y
K8521		12	c.2695C > T	p.R899X	Nonsense	34 y
K10327		12	c.2695C > T	p.R899X	Nonsense	19 y

New identified mutations are indicated by asterisks (*)

**Figure 2:**
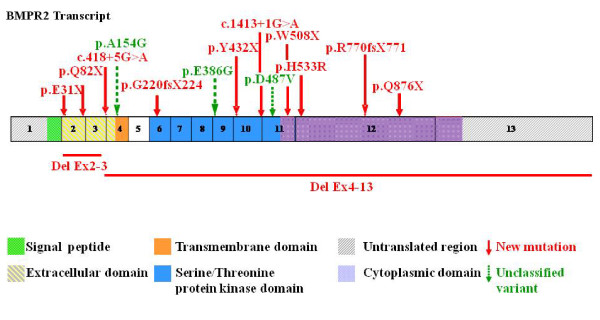
**Location of the new identified sequence alterations (mutations and/or unclassified variants)**. The figure shows the location of all newly identified mutations/unclassified variants through the *BMPR2 *transcript. Larger deletions are shown as line below the transcript, point mutations (nonsense and missense), splice site mutations and frameshift mutations are marked above the transcript as arrows, boxes represent exons, and colours of the boxes represent the different domains. Unclassified sequence alterations are highlighted in green and a dotted arrow. Mutations which are detected multiple times are only shown once. The mutations are widely distributed throughout the whole gene but two clusters are recognisable: cluster 1 lies in the extracellular domain (exons 2-4) whereas cluster 2 comprises exons 9 to 11 (serine/threonine protein kinase domain).

In 49 HPAH patients heterozygous alterations (46 mutations and 3 unclassified variants) in the *BMPR2 *gene were identified; 12 mutations and 3 unclassified variants have been detected for the first time in this study (Table 2, Figure 2). Table 2 lists all identified sequence alterations, the type of alteration, their location in the gene, and the age at diagnosis. New identified mutations or unclassified variants of the *BMPR2 *gene are indicated by asterisks.

#### Distribution and frequency of BMPR2 mutations

The 49 *BMPR2 *mutation types identified in the study population were: 35 point mutations (21 nonsense mutations and 14 missense mutations), 4 splice site mutations (all with affected splice donor sites), 4 frameshift mutations (small deletions/insertions) and 6 large deletions. The nonsense and the frameshift mutations resulted in a premature termination of the protein. The mutations were distributed throughout the whole *BMPR2 *gene with two clusters in a) the extracellular domain (exons 2-4) and b) the serine/threonine protein kinase domain (exons 9-11). Four mutations occurred in more than one independent patient/family: p.R491W and p.R321X three times, respectively; p.C420Y, p.R873X and p.R899X two times, respectively.

In 8 of the 209 apparently sporadic cases *BMPR2 *mutations have been identified and subsequently, thorough analysis of their family members revealed the same mutation in further asymptomatic members (in 3 parents, 5 children, 2 siblings), indicating that the proportion of sporadic PAH has been over estimated.

#### New mutations (Figure 2, Table 2)

Five of the 12 identified, to the best of our knowledge not yet described *BMPR2 *mutations were nonsense mutations, 2 frameshift mutations, 2 larger deletions, 2 splice defects, and one missense mutation. The *BMPR2 *mutations have been identified in exon 2-3, 6, 10, 11, and 12 (Table 2, Figure 2).

Three of the identified 14 missense mutations are unclassified sequence variants (p.E386G, p.D487V and p.A154G) (Table 2 and 3). Their disease causing potential has not been clearly verified. Analysis of these variants by use of the program MutationTaster [20] showed that all three variants are predicted to be most likely disease causing mutations (Table 3). The p.E386G and the p.D487V variants were both located in the serine/threonine kinase domain which is a highly conserved region among different species and suggests an important role in the function and/or structure of this region whereas the p.A154G variant was located at the beginning of the transmembrane domain. The variants were additionally analysed with the program ESEfinder [21,22] to investigate whether these substitutions might have an effect on exonic splicing. According to this analysis, the p.A154G and p.D487V variants had no effect on ESE binding sites whereas the p.E386G variant resulted in loss of 1 SF2/ASF- and 1 SRp40-site, respectively which might have an influence on the correct splicing [Table 3].

**Table 3:** Analysis of the Unclassified *BMPR2 *Sequence Variants by use of computer prediction programs

Unclassified variant	Localization	MutationTaster prediction	Conservation across different species	ESEfinder prediction	Clinical classification according to family history
p.A154G	Transmembrane domain	Disease causing	conserved	Not affected	IPAH

p.E386G	Serine/threonine kinase domain	Disease causing	conserved	SF2/ASF-& SRp40-site affected	IPAH

p.D487V	Serine/threonine kinase domain	Disease causing	conserved	Not affected	PAH with familial history

### Clinical characterization of patients with familial PAH but no detectable mutation

**Figure 3:**
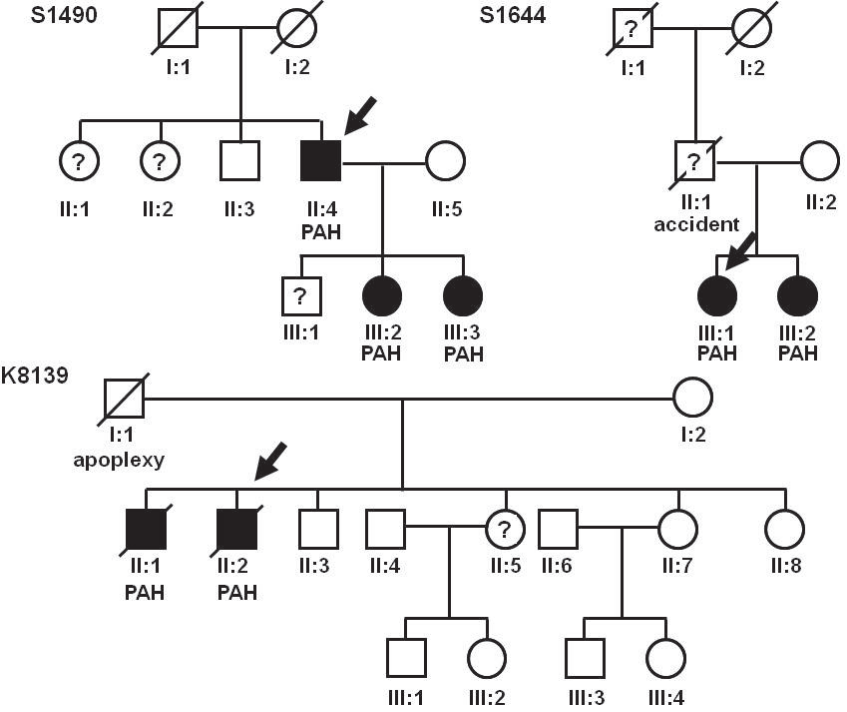
**Pedigree trees of familial PAH cases without mutation**. The figure represents pedigree trees of familial cases without mutation in *BMPR2*, *ACVRL1*, *ENG*, and *SMAD8 *(family S1490; family S1644 and family K8139). The index patient of each family is marked by an arrow. The father of the index patients in family S1644 also died quite young with an age of 47 due to an accident.

Only in 3 out of the 22 families with HPAH (13.6%, Figure 3) examination of the *BMPR2 *gene (promoter and coding regions including flanking intronic regions) and the coding regions of the *ACVRL1*, *ENDOGLIN*, and the *SMAD8 *genes did not reveal any defect. Especially, no point mutations or gross deletions/duplications were detectable.

In the affected members of all three families PAH has been diagnosed very early (mean age: 27.3 y ± 4.78) and was characterised by a very severe and rapid progressive clinical phenotype (mean hemodynamic values of the index patients catheterization at diagnosis were: mPAP: 56.3 ± 13.22 mmHg; PCWP: 7.7 ± 2.05 mmHg; CI: 2.01 ± 0.47; mean PVR 812 ± 68 dyn; heart rate 91.7 ± 9.43 beats/min). Although no mutations could be identified in the coding regions of the investigated genes there might be defects located in deeper intronic regions which could not be detected by conventional analysis methods or in other, until now, not identified genes participating in the *BMPR2 *signalling pathway.

### Family S1490

The male index patient (II:4) in this family presented first symptoms at age of 31 years and died early at an age of 33 years due to sudden right heart failure after an infection, two months after PAH was diagnosed. About 20 years later his children presented for familial screening assessment in Heidelberg. This analysis revealed a severe PAH in his two daughters (III:2, III:3). They have been early listed for double lung transplantation, which has been successfully performed 2 years after diagnosis.

### Family S1644

The female index patient (III:1) of this family was invasively diagnosed at an age of 22 years. She was severely affected with NYHA class III, severely impaired right ventricular function and hemodynamic values (heart rate per min: 85; mPAP: 75 mmHg; PCWP: 8 mmHg; CI: 2.0). Her sister (III:2) died very young (age 22 years) because of PAH, no DNA sample was available. She had dyspnoea from early childhood on and was initially diagnosed as bronchial asthma although no asthma attacks had occurred. The father also died quite young with an age of 47 years because of an accident and could not be examined. No other family members showed signs of PAH. Sequence and MLPA analysis were both negative for mutations or deletion/duplication in all investigated genes (Figure 3).

### K8139A

A rapid progressive clinical phenotype has been detected in this family as well. The male index patient (II:2) showed first symptoms of PAH at an age of 33 years which was finally confirmed by right heart catheterization at age of 34 years (heart rate per min: 105; pulmonary arterial systolic pressure: 68 mmHg; pulmonary arterial diastolic pressure: 36 mmHg; mPAP: 46 mmHg; PCWP: 5 mmHg; SASP: 85 mmHg; SADP: 60 mmHg; CI: 1.44; PVR: 744 dyn). He presented with NYHA class III-IV, severely impaired right ventricular function and died finally at the age of 38 years although he has received a triple PH-specific therapy including intravenous prostacyclin. He had refused the listing for lung transplantation. His affected older brother (II:1) died also very young (age 26 years) because of PAH within three months after appearance of the first symptoms. No DNA sample was available from him. The father (I:1) died at age of 68 years due to an apoplexy. The familial screening assessment revealed no PAH in any other family member so far (Figure 3).

## Discussion

In this study, we confirmed previous findings that *BMPR2 *mutation carriers are younger at diagnosis with a more severe hemodynamic compromise in a large prospectively assessed cohort of patients with confirmed PAH. Furthermore, we identified 12 to the best of our knowledge not yet described *BMPR2 *mutations and 3 unclassified sequence variants.

The study obtained *BMPR2 *mutations in 86.4% of HPAH patients with a positive family history and in 14.4% of patients with apparently sporadic disease. Only in 3 out of 22 families with confirmed HPAH (13.6%) no genetic defect could be detected. This result suggests that with the increasing knowledge on *BMPR2 *sequence alterations and the improving diagnostic genetic techniques the rate of identifiable genetic defects in familial PAH might be even higher ( > 80%) than previously suggested (≈ 70%) [1,7].

### BMPR2 mutations and clinical phenotype

Previous data indicated that having *BMPR2 *mutations is associated with a more aggressive form of PAH based on an earlier age at diagnosis and more severe hemodynamic [6,12-15]. Although survival was similar in mutation carriers and non-carriers, patients with *BMPR2 *mutation were more likely to be treated with parenteral prostacyclin therapy or to undergo lung transplantation [13]. Worse hemodynamic parameters [12] and reduced vasoreactivity [6,12,14-16] have been described in PAH-patients with non-synonymous *BMPR2 *mutations. The study performed by Rosenzweig et al [12] included children and showed a significant lower cardiac index but no significantly higher mPAP or PVR. No significantly differences in the hemodynamic parameters of mutation carriers vs. non-carriers have been found by Dewachter et al [23]. They suggested this might be due to the small number of patients (n = 28) in this study [23].

However, some studies have been retrospective in design. Our study has analysed the impact of *BMPR2 *mutations on the age at diagnosis and hemodynamic parameters for the first time in a prospective design and confirms the findings of the previous studies [6,12,14,15].

Due to the limited number of patients carrying a *BMPR2 *mutation most studies do not allow to sufficiently correlate distinct mutation types with clinical presentation. Austin et al. [17] showed that PAH patients carrying a truncating mutation in the *BMPR2 *gene developed a more severe disease than patients without truncating mutation. No correlation was seen in our data between truncating mutation and gender, age of onset, and hemodynamic values. This is in concordance with the results of the French PAH registry [15].

Since occurrence of *BMPR2 *mutations obviously influences the clinical phenotype genetic testing may become of increasing clinical relevance. Patients with *BMPR2 *mutation tend to a more severe clinical phenotype and might be followed more closely. Clinical assessment of family members [11,24] might be therefore especially of importance in patients with detected mutations.

### Identification of new *BMPR2 *mutations

In this study we identified different types of mutations resulting in a truncated protein which might all interfere with the downstream signalling of the BMP pathway (for example by nonsense mediated decay) and activate proliferating pathways [25]. The detected *BMPR2 *mutations were distributed throughout the whole gene with 2 clusters as described previously [1,6,15]. Cluster 1 was located in the extracellular domain (exons 2-4) whereas cluster 2 comprised exons 9 to 11 (serine/threonine protein kinase domain). As a consequence the complete gene should be genetically analysed in clinical routine.

From 49 mutations 12 were newly identified and were predominantly nonsense mutations. Three newly found missense mutations were termed unclassified variants because their disease causing potential has not been clearly verified until now. Analysis of these variants by usage of different prediction programs showed that all three variants are predicted to be most likely disease causing mutations (Table 3). Two of them (p.E386G and p.D487V) are located in the serine/threonine kinase domain which is a highly conserved region among different species and suggests an important role in the function and/or structure of this region whereas the third (p.A154G) variant is located at the beginning of the transmembrane domain. Therefore, all three variants are predicted to have an impact on the proper function of the protein.

### PAH families without *BMPR2 *mutation

Three of the 22 familial PAH cases without mutation in the *BMPR2 *gene investigated in our study did not reveal defects of the *ACVRL1 *gene, the *ENG *gene, and the *SMAD8 *gene. We have excluded them from genotype-phenotype analysis to reduce the risk of misclassification as has been described before [15]. Interestingly, mean age at diagnosis in this small group was even significantly lower as in all other patients. Girerd and collegues [14] described this for patients with familial PAH and hereditary hemorrhagic telangiectasia carrying a mutation in the *ACVRL1 *gene. In our families hereditary hemorrhagic telangiectasia and *ACVRL1 *gene defects have been excluded. The proportion of patients with familial aggregation but no detectable *BMPR2 *mutation was in our study even lower (13.6%) than in other cohorts (17.6% in the study performed by Austin et al. and 26.3% in the French registry, respectively [6,17]). Consequently, it may be assumed that mutations in the known genes *BMPR2*, *Activin A receptor type II-like 1, Endoglin*, and *SMAD8 *are not the only cause of the disease. However, in our 3 *BMPR2 *negative PAH families it is alternatively possible that these patients carry mutations in intronic or regulatory regions, which have not been detected by the used standard techniques.

Thus, in patients with familial aggregation of PAH *BMPR2 *mutations are most likely. Genetic testing including the complete *BMPR2 *gene may improve risk stratification in all patients with PAH.

## Competing interests

The authors declare that they have no competing interests.

## Authors' contributions

JSH and KH carried out the molecular genetic studies. NP carried out the molecular genetic studies, drafted the manuscript and evaluated the molecular genetic data. CF performed the statistical analysis and drafted the manuscript. NE, CN, HT, HO, FR, AHAG and EG treated the patients and collected data. EG and WS conceived of the study, and participated in its design and coordination and drafted the manuscript. All authors read and approved the final manuscript.
